# In vitro testing of salt coating of fabrics as a potential antiviral agent in reusable face masks

**DOI:** 10.1038/s41598-022-21442-7

**Published:** 2022-10-11

**Authors:** Sandra Schorderet Weber, Xavier Bulliard, Rosy Bonfante, Yang Xiang, Silvia Biselli, Sandro Steiner, Samuel Constant, Raphael Pugin, Alexandra Laurent, Shoaib Majeed, Stefan Lebrun, Michele Palmieri, Andreas Hogg, Arkadiusz Kuczaj, Manuel C. Peitsch, Julia Hoeng, Adrian Stan

**Affiliations:** 1 PMI R&D, Philip Morris Products S.A., Quai Jeanrenaud 5, 2000 Neuchâtel, Switzerland; 2 Centre Suisse d’Electronique et de Microtechnique SA (CSEM), Rue Jaquet-Droz 1, 2002 Neuchâtel, Switzerland; 3 Epithelix Sàrl, Chemin des Aulx 18, 1228 Plan-les-Ouates, Geneva, Switzerland; 4 Coat-X SA, Eplatures-Grise 17, 2300 La Chaux-de-Fonds, Switzerland

**Keywords:** Viral infection, Antivirals, Influenza virus, SARS-CoV-2, Disease prevention

## Abstract

During the coronavirus disease (COVID-19) pandemic, wearing face masks in public spaces became mandatory in most countries. The risk of self-contamination when handling face masks, which was one of the earliest concerns, can be mitigated by adding antiviral coatings to the masks. In the present study, we evaluated the antiviral effectiveness of sodium chloride deposited on a fabric suitable for the manufacturing of reusable cloth masks using techniques adapted to the home environment. We tested eight coating conditions, involving both spraying and dipping methods and three salt dilutions. Influenza A H3N2 virus particles were incubated directly on the salt-coated materials, collected, and added to human 3D airway epithelial cultures. Live virus replication in the epithelia was quantified over time in collected apical washes. Relative to the non-coated material, salt deposits at or above 4.3 mg/cm^2^ markedly reduced viral replication. However, even for larger quantities of salt, the effectiveness of the coating remained dependent on the crystal size and distribution, which in turn depended on the coating technique. These findings confirm the suitability of salt coating as antiviral protection on cloth masks, but also emphasize that particular attention should be paid to the coating protocol when developing consumer solutions.

## Introduction

In response to the coronavirus disease (COVID-19) pandemic and following the recommendations of the World Health Organization^1^, most countries have developed strategies and guidelines to control the transmission of severe acute respiratory syndrome coronavirus 2 (SARS-CoV-2) in the general population. In addition to social distancing, the guidelines encourage or mandate the use of face masks (either disposable or reusable) in public places^2^. This has led to a widespread use of face masks by the general population for curbing the transmission of respiratory diseases. The use of non-medical, reusable, cloth face masks^3^, whether homemade or commercially produced, was an accepted solution to the initial shortage of disposable face masks^4^ and was immediately recognized as a potential for increasing the sustainability of the public health guidelines that were established. The protection that reusable face masks provide against virus transmission depends on their filtration performance for particles that are 1–3 μm in diameter^5,6^, which greatly varies with the material used to manufacture them^7–11^. Above 3 μm, the filtration efficiency is usually at 100% for most common textiles^10^, resulting in the efficient blocking of any particles with a diameter > 3 μm that may come in contact with the mask. Because particles of this size may carry large virus loads^12^, coating the surface of the mask with an antiviral agent may reduce fomite transmission.

During the initial stages of the COVID-19 pandemic, concerns about the risk of self-contamination^1^ have fueled a debate on whether the untrained general population should be wearing face masks due to the fear of self-contamination offsetting the benefits of their use^13^. In this context, the scientific community strongly advocated wearing masks^14,15^, which was supported by studies^16,17^ and mathematical models^18,19^. Thus, coating the mask surface with antiviral agents may help reduce the indirect transmission of respiratory viruses^20–22^. Metals and metal oxides, antimicrobial polymers, photoreactive or carbon-derived material, and biomolecules have been considered as antiviral mask coatings^23–27^ and have been extensively reviewed^13,28,29^. Quan et al.^20^ described a simple method for coating surgical face masks made from non-woven (melt-blown) polypropylene with sodium chloride (NaCl). They reported that H1N1 influenza viral particles were inactivated by physical disruption of their capsid within 5 min due to local dissolution and recrystallization of the coated salt when wetted by virus-laden aerosols^20,30^. This coating was stable and maintained its efficacy even after storage in harsh conditions^20^. The authors later demonstrated that salt coatings functionalize inert membranes for the efficient capture and inactivation of airborne pathogens^31^. For use in antiviral coatings, NaCl is safe, inexpensive, easy to obtain, and could be used in non-professional environments for the production of both homemade and commercial reusable face masks.

The objective of the present work was to further evaluate the salt solution formula reported by Quan et al.^20^ for coating reusable face masks made from common fabrics. As cloth face masks are washed regularly for reuse, the users will have to renew the antiviral salt coating after each wash. Therefore, we investigated whether salt-deposition methods suitable for home use, i.e., spraying and dipping, and common fabrics recommended for the manufacturing of reusable face masks^10^ would affect the antiviral properties of the deposited salt layer.

We selected a universal household cleaning cloth as a test fabric and coated it with incremental concentrations of salt via automated controlled spray or dip application. The amount of salt deposited, and crystal size and distribution were assessed to characterize the coating conditions before virus exposure. The antiviral properties of the coatings were tested and compared by in vitro bioassays using influenza A H3N2 virus (A/H3N2, family *Orthomyxoviridae*, genus *Alfainfluenzavirus*, species *Influenza A virus*, serotype H3N2) cultured in a three-dimensional (3D) lung epithelial tissue culture model.

## Results

### Salt deposition methods impact the structure of the salt coatings

Five spray and three dip coating conditions were defined. Spray deposition with the salt formulation containing Tween-20 as wetting agent^20^ was performed, one piece of fabric per condition, using a spray device whose valve aperture was changed according to the arbitrary stroke units 1, 3, 5, and 10, labeled Spr S1, Spr S3, Spr S5, and Spr S10, respectively. The spray formulation was fivefold diluted for an additional sample with stroke unit 3 (Spr S3 Dil5 ×). Dip coating was performed on three pieces of material per condition with the non diluted (Dip No Dil), fivefold (Dip Dil5 ×), and tenfold diluted salt formulation (Dip Dil10 ×). The amount of salt deposited on the fabric (mg/cm^2^), the distribution and size of the salt crystals were measured to characterize each coating condition, before exposure to viral particles.

**Figure 1:**
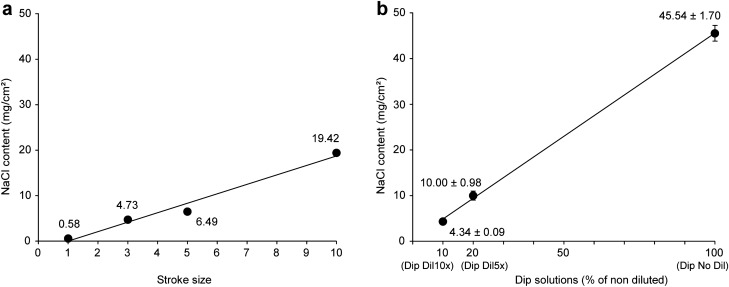
Concentrations of salt (sodium chloride, NaCl) deposited on spray- and dip-coated fabrics. For the Spr treatments, a single piece of material (42.25 cm^2^) was prepared, and three pieces (1 cm^2^ replicates) were cut out of it. For the Dip treatments, three samples (7.5 cm^2^) were prepared separately. (**a**) Spray coating with stroke units 1, 3, 5, and 10. Regression line equation: Y = 2.0805x − 2.076; R^2^ = 0.9774; R = 0.9886; *P* < 0.05. (**b**) Dip coating with a salt solution diluted 10 × , 5 × , or no dilution. Data are means ± standard deviations. Regression line equation: Y = 45.256x + 0.3492; R^2^ = 0.9968; R = 0.9984; *P* < 0.0001. Weighed salt concentrations are displayed and expressed in milligrams per square centimeter.

The spray and dip coating methods yielded a linear increase of salt deposited on the fabrics relative to the stroke unit applied or dilution of the salt concentration of the dip solution (Fig. 1).

**Figure 2:**
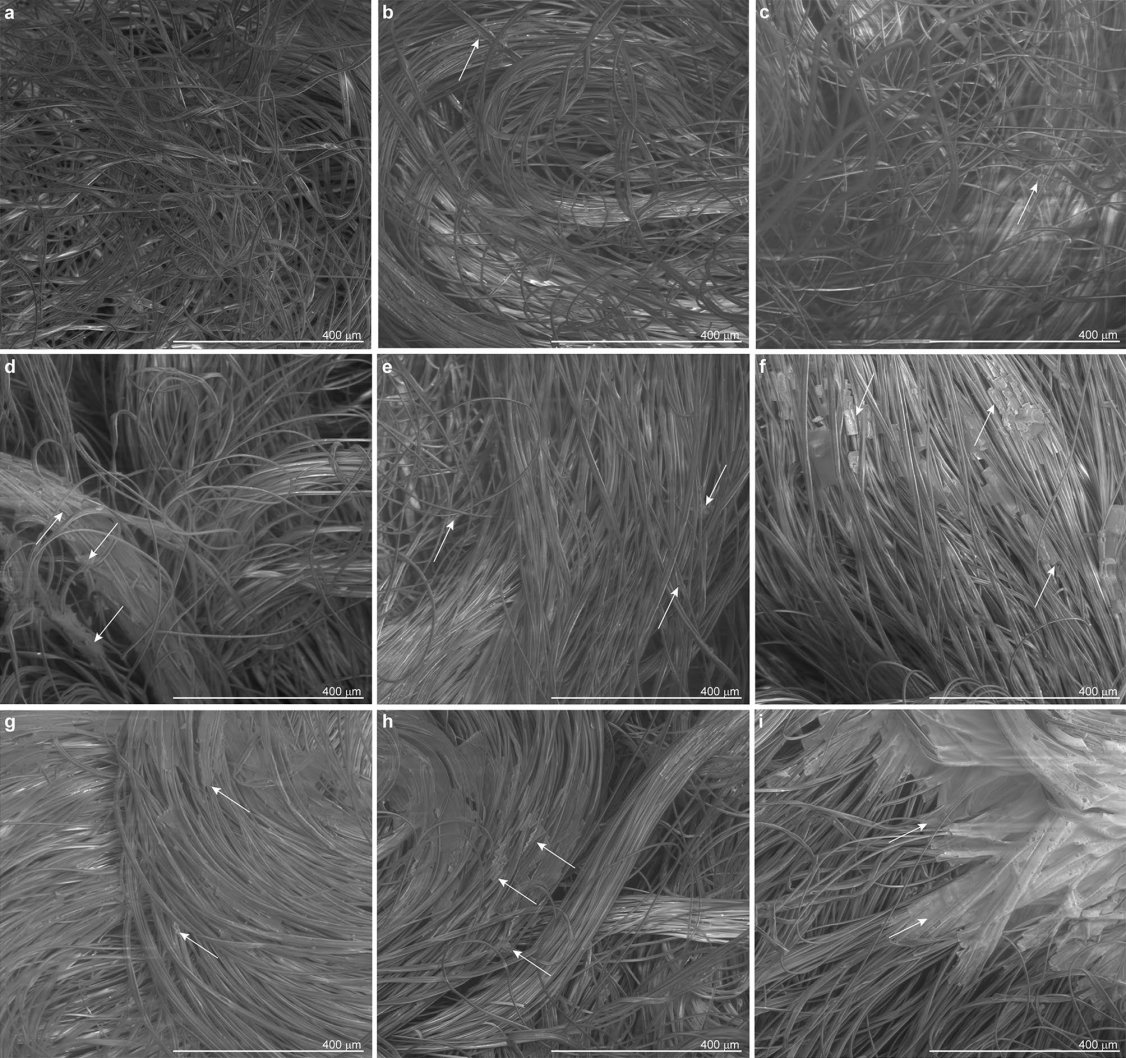
Scanning electron microscopic images of test material, salt-coated by spray and dip methods. The salt formulation was used non-diluted or diluted. Spray treatments allowed the deposition of increasing amounts of salt by varying the valve aperture of the spray device according to arbitrary stroke units. (**a**) Non-coated, (**b**) spray, undiluted salt formulation, stroke unit 1 (Spr S1), (**c**) spray, fivefold diluted salt formulation, stroke unit 3 (Spr S3 Dil5 ×), (**d**) spray, undiluted salt formulation, stroke unit 3 (Spr S3), (**e**) spray, undiluted salt formulation, stroke unit 5 (Spr S5), (**f**) spray, undiluted salt formulation, stroke unit 10 (Spr S10). For dip treatments test materials were immersed into (**g**) tenfold diluted (Dip Dil10 ×), (**h**) fivefold diluted (Dip Dil5 ×), and  (**i**) undiluted (Dip No Dil) salt formulations. Arrows on images point at examples of salt crystals and aggregates found on the test materials.

Scanning electron micrographs of test material (Fig. 2) show that following deposition and evaporation, both spray- and dip-coating methods produced a distributed scatter of crystals along the fabric fibers.

The crystal size was determined by the spray flow rate in the spray-coating method and by the dilution rate of the dip solution in the dip-coating method. With the spray-coating method, low flow rates resulted in small, monodisperse crystals, whereas at higher flow rates, larger, more polydisperse crystals were formed following the drying of the coalesced liquid deposited on the fabric surface.

The non-coated sample (Fig. 2a) and the samples with the lowest deposited volumes, Spr S1 (Fig. 2b) and Spr S3 Dil5 × (sprayed with a higher volume, but a lower salt concentration;  Fig. 2c), had a similar appearance. Very few salt crystals were observed scattered on the fibers of the Spr S1 and Spr S3 Dil5 × samples. However, although not visible by scanning electron microscopy (SEM), salt crystals were present on the fibers of the Spr S1 and Spr S3 Dil5 × samples as confirmed by energy-dispersive X-ray spectroscopy (EDX) (Supplementary Fig. S1a and b, respectively). For the medium spray volumes, Spr S3 and Spr S5, salt crystals were more clearly visible on scanning electron micrographs; they formed small aggregates along the fibers, more distinctly in Spr S3 samples than in Spr S5 samples (Fig. 2d,e, respectively). At the highest spray volume, i.e., Spr S10 samples (Fig. 2f), large crystals that enveloped several fibers were formed. In Spr S3, Spr S5, and Spr S10 samples, in addition to the large crystals observed by SEM, small salt crystals were distributed along the fibers throughout the material as revealed by EDX (Supplementary Fig. S1c–e).

In the dip-coating method, both diluted (Dip Dil10 × and Dip Dil5 ×) and non-diluted (Dip No Dil) solutions (Fig. 2g–i, respectively) led to the formation of salt crystals that covered the fibers. As in the sprayed test samples, the average crystal size on dip-coated fibers varied with the salt concentration in the solution. The Dip Dil10 × sample showed moderate-sized salt crystals (Fig. 2g), whereas the Dip No Dil sample was characterized by very large salt crystals that completely covered the fibers (Fig. 2i). Test materials heavily coated with salt, i.e., Spr S10 and Dip No Dil samples, became very stiff during handling. Such stiffening was not observed at lower salt concentrations.

### Salt coatings slow down virus driven epithelial structure disruption in MUCILAIR

After direct contact with the coated samples, viral particles were collected and used to inoculate MUCILAIR human airway epithelial cell culture inserts. The same protocol was run with fabric samples spray-coated with a 1% Tween-20/water solution with strokes 3, 5, and 10 (Tween Str3, Tween Str5, and Tween Str10). Epithelial integrity during the course of the viral infection was monitored by measuring transepithelial resistance (TEER) in comparison with non-treated uninfected cultures (Mock) and directly infected cultures (no-mask). TEER is a dynamic and non-invasive measurement that reflects the integrity of epithelium and typically ranges from 200 to 600 Ω·cm^2^ in undamaged MUCILAIR cultures^32^. Disruption of cellular junctions and presence of holes in the epithelia result in TEER values below 100 Ω·cm^2^.

**Figure 3:**
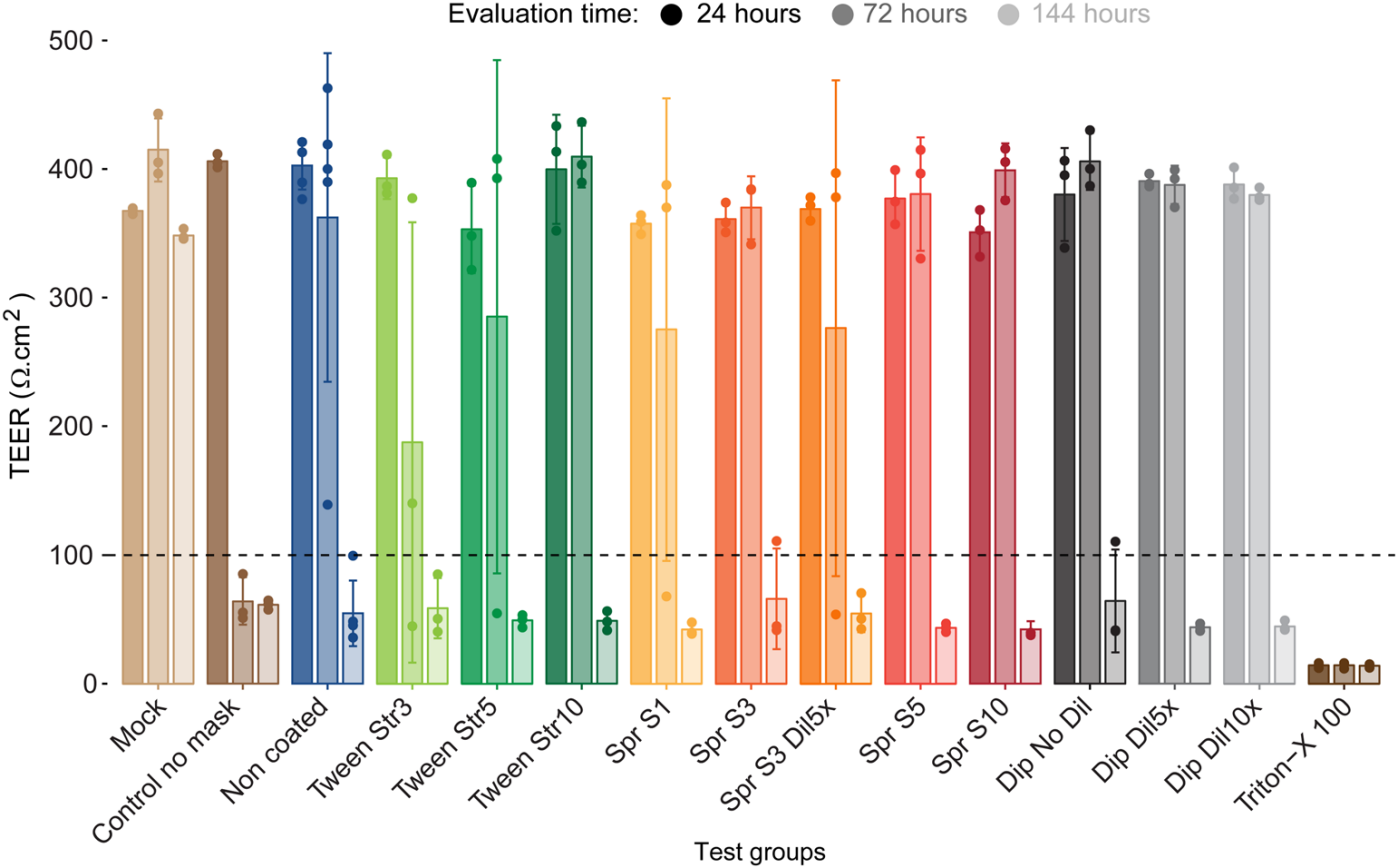
Transepithelial electrical resistance (TEER) measurements in MUCILAIR epithelia at 24, 72, and 144 h post infection. Means ± standard errors are shown; n = 3 in all treatments, except for non-coated samples (n = 5). The dotted line represents the 100 Ω·cm^2^ limit of tissue integrity. Mock, non-infected non-treated control culture; Triton X-100 detergent used as a positive control.

Twenty-four hours after viral infection, all MUCILAIR epithelia showed TEER values comparable with that of the mock control (Fig. 3). At 72 h post infection (hpi), TEER values decreased to below 100 Ω·cm^2^ under the no-mask control condition indicating severe disruption of the epithelial structure due to intense viral replication. On average, all cultures inoculated with virus collected after incubation with the treated test materials retained TEER values comparable with that of the mock control. However, under test conditions with the lowest concentrations of salt (Spr S1 and Spr S3Dil5 ×) and Tween-20 (Tween Str3 and Tween Str5), one replicate culture in each treatment group had a TEER value below 100 Ω·cm^2^. In contrast, in the test conditions with higher salt and Tween-20 concentrations, all replicate cultures had TEER values similar to those of the mock control. At 144 hpi, tissue integrity was severely affected by viral infection in all cultures, regardless of the test conditions.

### Exposure to salt and Tween-20 coatings impacts viral replication in MUCILAIR epithelia

A comparative analysis of virus replication in cells could only be performed 24 and 72 h post-infection; viral genome copies were not detectable in any of the samples before 24 h. Beyond 72 hpi, viral replication in cells markedly decreased in the no-mask control condition and reached a plateau in the non-coated sample, biasing the comparison between the treatments (data not shown), as has been previously encountered with this epithelial system^32^.

**Figure 4:**
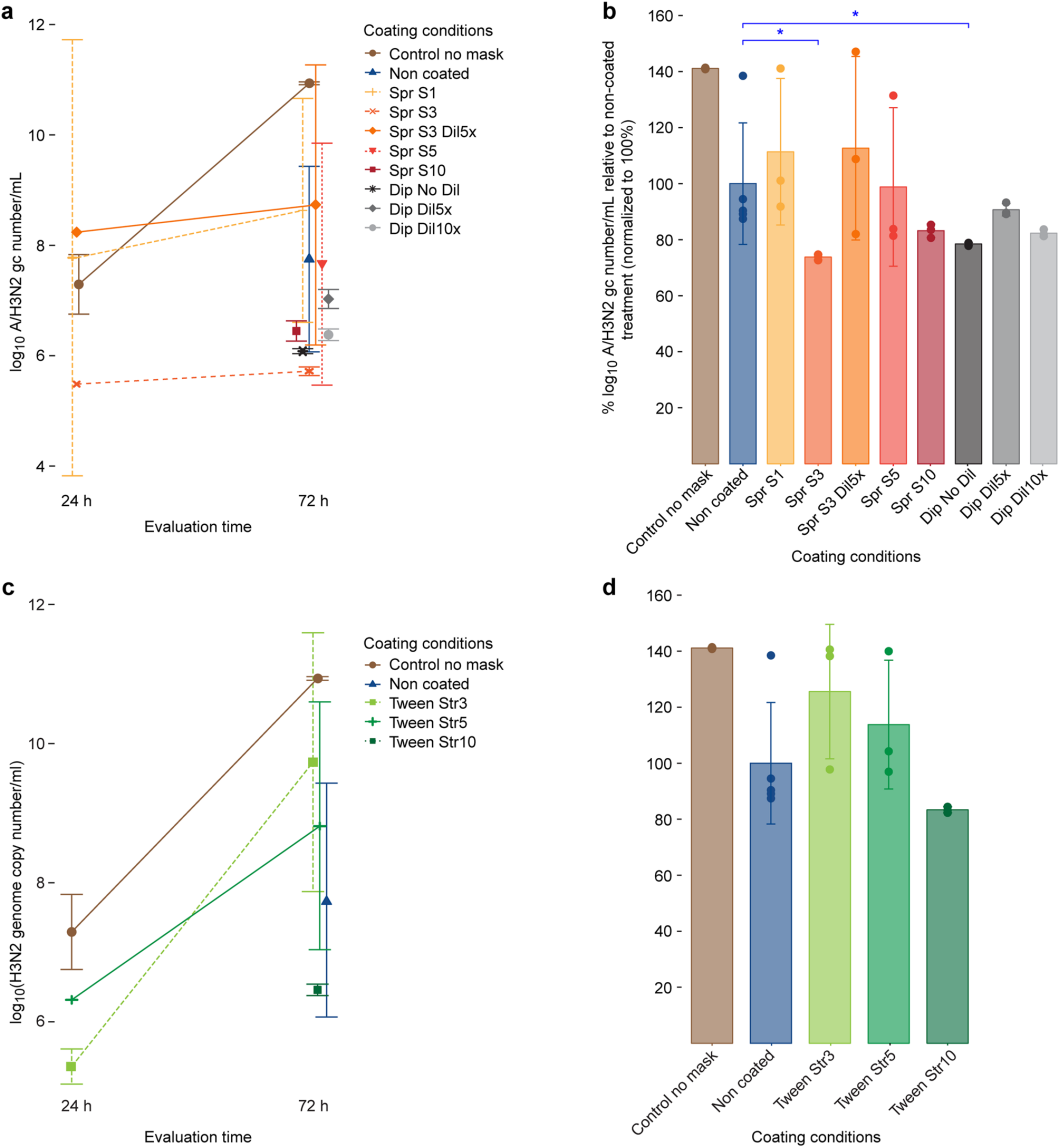
TAQMAN reverse transcription-polymerase chain reaction for the determination of A/H3N2 virus genome copy (gc) numbers in the apical medium of MUCILAIR epithelia infected after direct contact of the virus with a non-coated test material or a test material coated with various concentrations of salt or Tween. (**a**) Salt coatings, log_10_ A/H3N2 gc number/mL quantified at 24 and 72 h post infection. (**b**) Salt coatings, viral replication at 72 h post infection. (**c**) Tween coatings, log_10_ A/H3N2 gc number/mL quantified at 24 and 72 h post infection. (**d**) Tween coatings, viral replication at 72 h post infection. Data are expressed as a percentage relative to the mean of log_10_ A/H3N2 virus gc number/mL quantified in the infected non-coated treatment group, which was normalized to 100%. Means ± standard errors are shown; n = 3 in all treatments, except for non-coated (n = 5). *P < 0.05.

At 24 hpi, viral genome copies could be detected and quantified in cell apical washes collected from the no-mask control and the samples treated with the lowest salt (Spr S1, Spr S3Dil5 × , and Spr S3) and Tween-20 concentrations (Tween Str3 and Tween Str5), but not from the other samples (Fig. 4a,c).

Seventy-two hours after the infection, virus was detected in apical washes from all coating treatment groups. However, all treatments, including no coating (non-coated), affected viral replication as reflected by a decrease in viral genome copy numbers in the cell-culture apical washes when compared with that in the infected control culture (no-mask control, Fig. 4b). Compared to the non-coated condition, salt coating under the Spr S3, Spr S10, Dip No Dil, and Dip Dil10 × treatments exerted an additional, marked antiviral effect, significant for Spr S3 (*P* = 0.0269) and Dip No Dil (*P* = 0.0454). The lowest numbers of A/H3N2 genome copies were obtained in apical washes collected from the Spr S3 and Dip No Dil cultures, which exhibited log_10_ reductions of 4.31 and 3.95, respectively, in the genome copy numbers compared to those in the infected, non-coated treatment cultures. Although not statistically significant, the decrease in viral replication under Spr S10, Dip Dil 5 × , and Dip Dil treatments was still remarkable, with log_10_ reductions of 3.5, 2.99, and 3.65, respectively. We observed no clear correlation between the salt concentration in the coating and virus genome copy levels, as the best results were obtained with Spr S3 and Dip No Dil samples, with salt concentrations of 4.73 and 45.54 mg/cm^2^, respectively. A relevant antiviral activity seemed to be achieved above a certain threshold of salt deposits on the material surface. The Spr S5 treatment was the only exception to this observation, which could have been expected to result in a more prominent reduction in genome copy numbers. Nevertheless, two out of the three replicates exhibited marked log_10_ reductions of 3.74 and 3.54 in genome copy numbers, whereas only one replicate did not show an effect on viral replication.

In the Tween-20 test conditions (Fig. 4c,d), only the highest concentration of Tween-20, i.e., the Tween Str10 treatment, markedly affected viral replication, with a log_10_ reduction of 3.57 in the genome copy numbers compared to those of the non-coated test samples.

### Infectivity of A/H3N2 virus particles collected from MUCILAIR epithelia is reduced after contact with salt coatings

**Figure 5:**
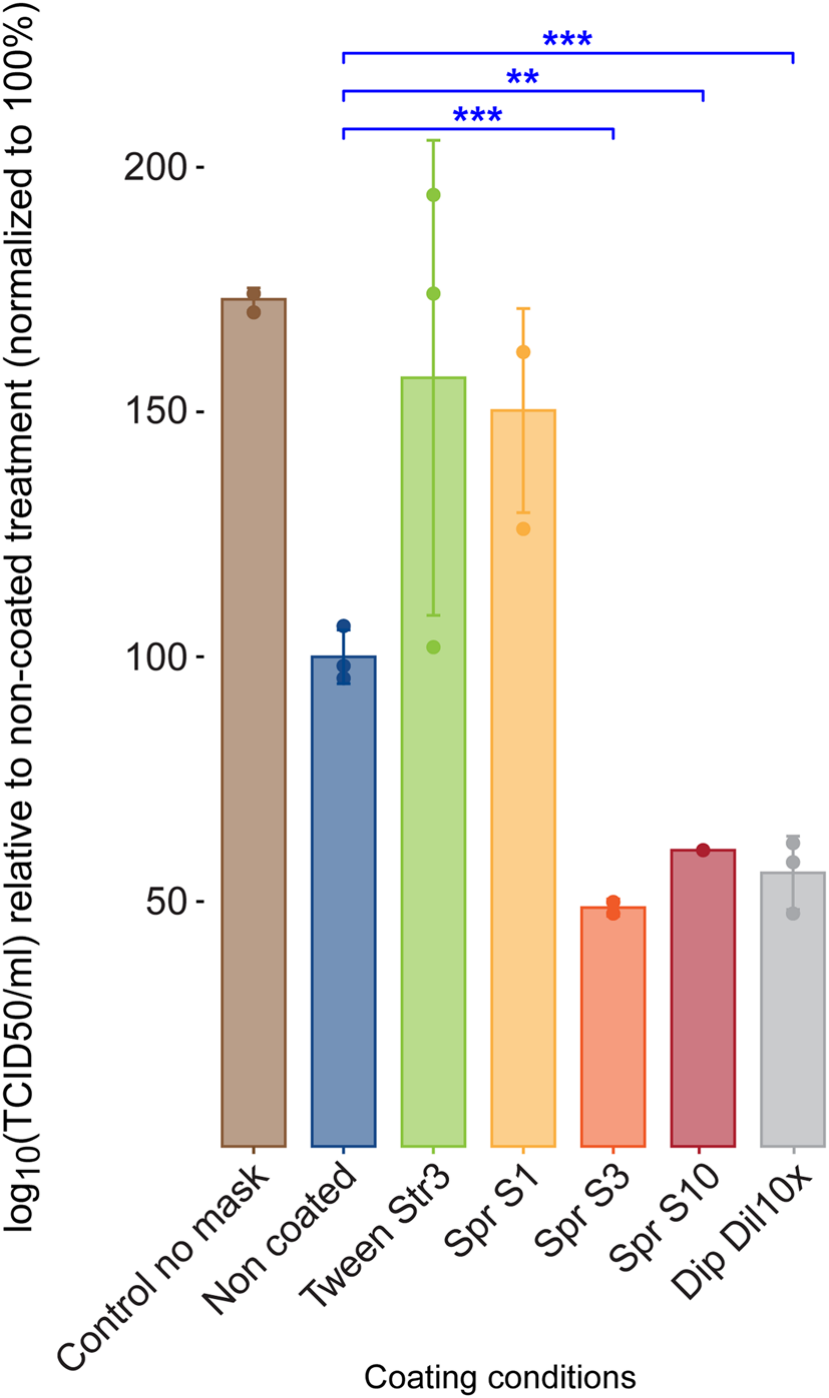
A/H3N2 virus titers in the apical medium of MUCILAIR epithelia infected after direct contact of the virus with a non-coated test material or test material coated with various concentrations of salt, determined at 72 h post infection. Data are expressed as a percentage relative to the mean of log_10_ TCID_50_/mL in the infected non-coated treatment group, which was normalized to 100%. Means ± standard errors are shown; n = 3. **P < 0.01; ***P < 0.001.

Because the virus genome copy quantification in cell apical washes may not be a full indicator of the infectivity potential of the viral particles, we determined the 50% tissue infectivity dose (TCID_50_) for selected test conditions (Fig. 5) by performing cell-based virus titrations in Madin-Darby canine kidney cells transfected with cDNA encoding human α-2,6-sialyltransferase (MDCK-SIAT1).

As observed previously for genome copy numbers, virus titers were significantly lower under test condition Spr S3 (*P* = 0.000752), and also under conditions Spr S10 (*P* = 0.00318) and Dip Dil 10 × (*P* = 0.000856), than under the non-coated test condition. The TCID_50_ assay results were in accordance with those obtained by quantitative reverse-transcription polymerase chain reaction (RT-qPCR), which indicated a marked decrease in the number of virus particles that were able to survive and replicate after 10 min of direct contact with the salt-coated fabrics.

### Pre-incubation of the virus in hypertonic salt solutions does not reduce virus infectivity

**Figure 6:**
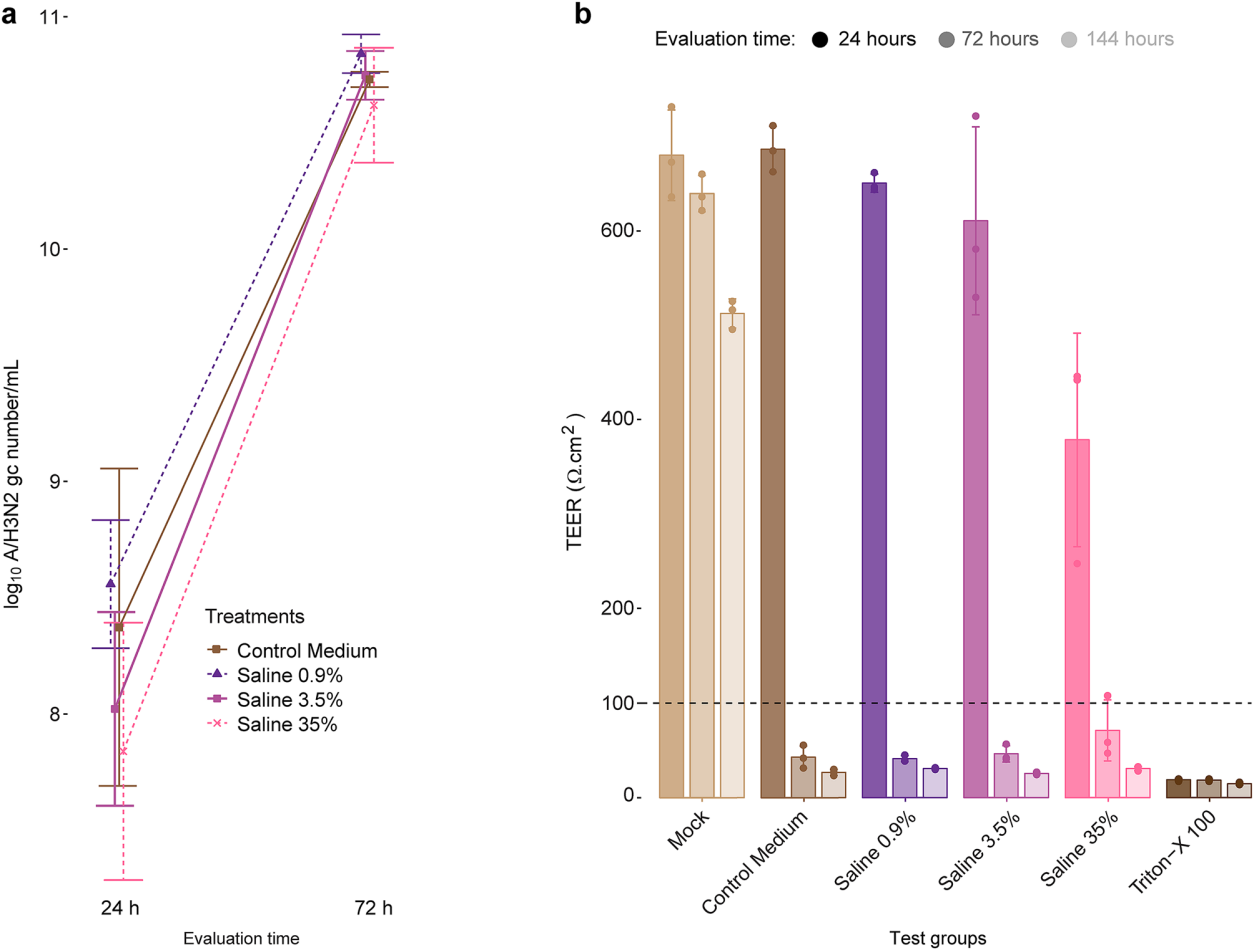
Effect of virus pre-incubation in hypertonic salt solutions on viral replication in cells. (**a**) TAQMAN reverse transcription-polymerase chain reaction for the determination of A/H3N2 virus genome copies (gc) in the apical medium of MUCILAIR epithelia infected after incubation of the virus in salt solutions of various concentrations. Data are expressed as log_10_ A/H3N2 gc number/mL, quantified at 24 and 72 h post infection. Means ± standard errors are shown; n = 3. (**b**) Transepithelial electrical resistance (TEER) measurements in MUCILAIR epithelia 24, 72, and 144 h post infection. Means ± standard errors are shown; n = 3. The dotted line represents the 100 Ω·cm^2^ limit of tissue integrity. Mock, non-infected non-treated control culture; Triton X-100, detergent used as a positive control.

To collect virus particles, the inoculated test material was incubated for 5 min in cell medium. Although the virus-containing solution was diluted 1:10 with cell medium prior to cell infection, salt content was still above isotonicity in most cell samples (Supplementary Table S1) and may have affected virus penetration into the cells. To verify that the virus deactivation was solely due to direct contact of the viral particles with the salt-coated fabrics, and not taking place during the collection step or the MUCILAIR epithela inoculation phase, we incubated A/H3N2 virus particles in 0.9%, 3.5%, and 35% saline solutions. Viral infectivity was not affected by the treatment and viral genome copies could be quantified as early as 24 hpi (Fig. 6a), under all test conditions.

At 72 hpi, genome copy numbers increased by a log_10_ factor of 2–3 in the three saline test conditions, similar to that in the control medium. The TEER results supported that the saline treatments were ineffective in controlling the viral infection. As in the control medium, the TEER values dropped to below 100 Ω·cm^2^ as early as 72 hpi in the three saline test groups (Fig. 6b). In all samples, the epithelial integrity was rapidly disrupted due to extensive viral replication, even after incubation of the virus in a saturated salt solution (saline 35%) for 10 min.

## Discussion

The present study extended the findings of Quan et al.^20^ on material that has good particle filtration properties, that can be washed and integrated into reusable face masks^10^. In addition, we used salt coating techniques applicable in a household setting. Finally, we verified the antiviral property of the salt coating with a simple laboratory setting, in human non-modified 3D airway epithelia^33,34^. This testing model could be applicable to other types of antiviral coating on various materials.

The two salt-deposition methods, spray and dip-coating, led to the formation of crystals on the fabric surfaces, with the salt crystal sizes being dilution- and volume-dependent. Except for the lowest-concentration coating conditions, Spr S1 and Spr S3 Dil5 × , which were ineffective although the salt was well distributed on the fabric samples, all other test conditions resulted in a clear effect of the salt deposits on viral activity. In those samples, viral genome copy numbers were very low or not detectable 24 hpi. Viral replication was still reduced by 3–4 log_10_ 72 hpi in five test conditions, i.e., Spr S3, Spr S10, Dip No Dil, Dip Dil5 × , and Dip Dil10 × . These results were confirmed by the TCID_50_ assay results showing that fewer virus particles were able to infect cells after having been in direct contact with the salt-coated fabrics. These findings are in line with those of previous studies that demonstrated the effects of salt deposition on the inner filter in surgical masks followed by influenza A/H1N1 virus inoculation^20^, and salt spraying on the outer layer of surgical masks and subsequent inoculation with a pig virus (transmissible gastroenteritis virus)^35^. However, there was no clear negative correlation between the salt concentrations on the fabrics and the viral genome copy numbers in cell cultures. The strongest reduction in virus particles was observed for two very different salt coating concentrations, Spr S3 (4.73 mg/cm^2^) and Dip No Dil (45.54 mg/cm^2^). The other coating conditions in this range suppressed viral replication at lower levels, but still decreased viral load by a log_10_ factor of 3. In contrast, the Spr S1 (0.58 mg/cm^2^) and Spr S3 Dil5 × (0.41 mg/cm^2^) treatments were clearly insufficient for controlling viral replication, and we suggest that a threshold salt concentration is needed to inhibit viral replication. It should be noted here that the effective amounts of deposited salt reported by Quan et al.^20^ were between 6.16 mg/cm^2^ and 24, 64 mg/cm^2^, which is well within the range of salt amounts deposited on our active samples.

The antiviral effect of a salt coating appears to be a consequence of virus capsid disruption from local salt dissolution followed by recrystallization^20,30^. This effect may vary not only with the amount of salt in the coating, but also with the distribution and size of the salt crystals on the fabrics, which in turn depends on various salt-deposition parameters and techniques. This may explain the poor negative correlation observed in this study between the salt concentration on the fabrics and viral deactivation above a certain salt concentration threshold.

In parallel to the salt dissolution/recrystallization hypothesis, we considered the possibility that salt residues dissolved during the virus collection step may also have contributed to viral deactivation because of the hypertonicity of the medium, as previously shown for airborne microorganisms^35^. To test this hypothesis, A/H3N2 virus particles were incubated directly in solutions of varying salt concentrations, from isotonicity to saturation, prior to their inoculation in the MUCILAIR cell cultures. The results revealed that the saline solutions had no effect on viral replication over time and did not contribute to retaining epithelial cell integrity (TEER) compared to that in the control medium. In the fabric coating experiments, the salt concentrations in the virus collection step were clearly within the range of the saline solutions tested (0.95–5.46% for coating versus 0.90–17.9% for saline solutions; Supplementary Table S1). Similarly, in a test system involving SARS-CoV-2, pre-incubation of the virus with saline solutions up to 1.7% did not alter viral replication in Vero cells^36^, and incubation of A/H3N2 virus in 1 M (5.8%) NaCl solution for up to 1 h did not decrease hemagglutinin titers and plaque-forming units in MDCK-SIAT1 cells compared to that in a phosphate-buffered saline control^37^. These results confirmed that the incubation of the virus particles on the coated fabric alone was responsible for viral deactivation, as also recently demonstrated by Rubino et al.^30^. The inactivation effect may be attributed to the crystal-forming properties of the salts rather than the type of salt used, as other salts, including potassium chloride (KCl), potassium sulfate (K_2_SO_4_)^30^, and sodium dihydrogen phosphate (NaH_2_PO_4_)^37^, have been shown to produce a similar effect when coated on surgical masks.

In addition to mechanical capsid disruption due to salt dissolution/recrystallization, increased osmotic stress during salt recrystallization has been suggested to lead to the deformation and damage of the virus capsid, resulting in deactivation^20,30,38^.

Because the salt solution contained 1% Tween-20, we investigated whether this surfactant may have also played a role in viral deactivation. Tween-20 is a mild surfactant that can break lipid–protein interactions and solubilize membranes^39^, and hence, can affect the capsid of influenza A virus, which is an assembly of proteins embedded in a lipid membrane^40^. In the present study, three spray concentrations based on stroke sizes, which were also used in the salt coating method, i.e., strokes 3, 5, and 10, were tested. Only the highest concentration of Tween-20, in the Tween Str10 treatment, markedly affected viral replication in MUCILAIR cells with a log_10_ reduction of 3.57. This shows that Tween-20 may also have contributed to viral deactivation in the samples with the highest salt concentrations. In line herewith, Tween-20 was reported to reduce the survival of herpes virus at a concentration of 1%^41^ or 0.25%^42^.

Irrespective of these technical considerations, the findings of the present study confirm that salt can form a protective antiviral barrier not only on surgical masks, but also on household materials that could be used in washable cloth masks. Since the antiviral effect of salt was engendered by the physical disruption of the viral capsid, it is reasonable to assume that other enveloped viruses, such as SARS-CoV-2, could be affected as efficiently even if they are not closely related to A/H3N2. This cross-specific antiviral activity linked to capsid alteration has been reported for SARS-CoV-2 (positive-sense single-stranded RNA) and bacteriophage phi6 (double-stranded DNA) after contact with non-woven spunlace fabric coated with cranberry extract^26^. The current model with Influenza A/H3N2 and MUCILAIR human nasal epithelium can be implemented in a wide range of institutions as it only requires biosafety level two (BSL-2), while using SARS-CoV-2 would require BSL-3. This model that uses human nasal epithelia could be further extended to the evaluation of other antiviral coating and for various types of personal protective equipment, keeping in mind that the interactions between virus and antiviral coating might be over simplified and that positive results should be confirmed with a more sophisticated system involving aerosolized viral particles.

For coating reusable cloth face masks, both salt-deposition methods used in this study can be adapted for home use for renewing the protective salt layer on the mask after washing. Particular attention should be given to using a sufficiently concentrated salt solution to reach the antiviral activity threshold, while avoiding excessive loads of salt that render the mask stiffer and less comfortable, as observed for the Spr S10 and Dip No Dil coatings. However, breathability and particle filtration properties which are important parameters for the choice of the material to manufacture reusable face masks^10^ would probably hardly be affected by salt coating in the range of salt amounts used in the present study, and as previously demonstrated with large-pore polypropylene membranes coated with close concentrations of NaCl, KCl, or K_2_SO_4_^31^. Easy-to-purchase surfactants, such as hand soap, could effectively replace Tween^43^. Ready-to-use salt spray solutions may become a convenient option for achieving an optimal salt coating with adequate salt crystal size and distribution. A 10% salt spray solution device has been recently successfully tested^35^.

## Materials and methods

### Test material

The test material was a universal non-woven microfiber cloth made of 80% polyester and 20% nylon (JEMAKO Pro Cloth Plus, Jemako, Rhede, Germany). This material, widely available in retail stores, exhibits good particle filtration properties and breathability^9,10,44^.

### Coating solutions

The salt solution was composed of 29.03% w/v (29.03 g/100 mL) NaCl (Merck, Darmstadt, Germany) in deionized water with 1.0% v/v (1 mL/100 mL) Tween-20 (Merck), as reported by Quan et al.^20^. The salt solution was tested in non-diluted and diluted forms (5- or tenfold dilution in deionized water). Tween-20, a nonionic surfactant, was added to the salt solution for proper wetting of the hydrophobic polyester fibers^20^. To assess the effect of Tween-20, a 1.0% v/v (1 mL/100 mL) solution of Tween-20 in deionized water was prepared.

### Coating procedures

We used two automated coating procedures: spraying and dipping. Spray-coating deposition was achieved using a mini spray valve (EFD 781; Nordson, Westlake, OH, USA) mounted on an automated robot (JR2304; Janome, Tokyo, Japan). This system enabled control of the total amount of salt deposited by adjusting parameters, including the speed of deposition (set to 40 mm/s), the distance between the spray head and substrate (set to 40 mm), and the pressure applied on the cartridge containing the salt solution (set at 0.4 bar). The deposition pattern consisted of straight parallel lines 4 mm apart. The stroke (valve aperture controlling the flow) was varied with the arbitrary stroke units 1, 3, 5, and 10, labeled Spr S1, Spr S3, Spr S5, and Spr S10, respectively. To assess the effect of dilution at constant volume, an additional sample with stroke unit 3 was prepared with a fivefold-diluted coating solution (Spr S3 Dil5 ×). One piece of test material per condition sized 6.5 cm × 6.5 cm was treated.

Dip coating was performed using an automated dip coater (KSV Nima (medium size); Biolin Scientific, Espoo, Finland). Three pieces of test material per condition, sized 2.5 × 3 cm, were prepared. Dip coating was performed using three salt concentrations: not diluted, diluted 5 × , and diluted 10 × (labeled Dip No Dil, Dip Dil5 × , and Dip Dil10 × , respectively). The fabric pieces were fully immersed in the solution for 3 s and withdrawn at a constant speed of 100 mm/min. The pieces were suspended vertically and allowed to drain for 30 min.

To assess the potential antiviral effect of Tween-20, a 1% Tween-20/water solution was sprayed onto the test material using the above spraying device and conditions. Spray strokes 3, 5, and 10 were applied, labeled Tween Str3, Tween Str5, and Tween Str10, respectively. The samples were dried, stored, and handled in the same way as the salt-coated samples.

After coating, the material was dried at room temperature (20 °C) overnight and stored in clean, sealed plastic bags under a nitrogen atmosphere. For all salt-coated samples, the deposited salt was quantified by weighing the sample before coating and after drying. To determine the salt crystal size and distribution on the materials, the coated samples were analyzed by SEM and EDX. The protocols are described in the Supplementary Information.

### Epithelial tissue cultures

MUCILAIR-HF human airway cell models (Epithelix, Geneva, Switzerland) are fully mature, functional, 3D airway epithelia cocultured with fibroblasts. MUCILAIR epithelia (batch number HF-MP0009) were reconstituted with nasal epithelial cells isolated from 14 donors undergoing surgical nasal polyplectomy (age, 24–58 years, median, 53 years) and co-cultured with human fibroblasts on TRANSWELL inserts (Corning, Glendale, AZ, USA)^32^. All experimental procedures to obtain the cell samples were explained in full, and all subjects provided written informed consent. The study was conducted according to the declaration of Helsinki on biomedical research (Hong Kong amendment, 1989), and the research protocol was approved by the local ethics commission. Mature cultures (66-day-old) were used in the antiviral experiments. The cell cultures were quality-checked before use as described in the Supplementary Information.

### A/H3N2 virus stock preparation

A/H3N2 virus particles were originally isolated directly on MUCILAIR epithelia from a clinical specimen^33^, and the lineage, A/Switzerland/8004462/2013(H3N2), was identified by PCR, a hemagglutination inhibition assay, and partial sequencing of the neuraminidase gene. Stocks of this isolate were produced in MUCILAIR cultures by collecting apical washes with culture medium and used successfully in infection experiments^32,33^. The full production procedure is described in the Supplementary Information.

### Assessment of antiviral activity

Immediately before virus exposure, the coated material was removed from the bags at room temperature (26% relative humidity), cut into 1 cm^2^ pieces using scissors, and placed in sterile Petri dishes. All operations were performed in a clean and dry environment. In addition, 1 cm^2^ pieces of the uncoated material were prepared to serve as control samples. Both sides of the fabric pieces were sterilized by ultraviolet-C radiation for 30 min, using a UV900 G30T8 lamp (at 12.0 W for 100 h; Philips Lighting, Lamotte-Beuvron, France).

Prior to viral inoculation, the MUCILAIR epithelia inserts were placed into 24-well plates containing 500 µL/well of MUCILAIR culture medium, and washed with 200 µL of MUCILAIR culture medium at 34 °C for 10 min. Three pieces of test material per coating condition and five pieces of non-coated material, sized 1 cm^2^ each, were placed in separate wells of a 24-well plate (CORNING COSTAR 3526; Corning, NY, USA). From the virus stock, a solution containing 2 × 10^8^ genome copies were prepared in MUCILAIR culture medium. Five microliters of the A/H3N2 preparation (10^6^ gc) was deposited onto each piece of the test material. After 10 min of direct exposure, 1 mL of MUCILAIR medium was added to each well containing a test sample for collecting the virus. After 5 min incubation at room temperature, followed by mixing by pipetting movements, the medium containing the virus particles was transferred to a new 24-well plate and diluted 1:10 in MUCILAIR medium. Then, 100 µL of the solution was applied apically to cells grown on MUCILAIR inserts for infection at 34 °C, 5% CO_2,_ and 100% relative humidity for 3 h. The virus inoculum was then discarded and the cells were washed three times with 200 μL of MUCILAIR medium. At 3.5, 24, 72, and 144 h post viral inoculation, 200 μL of MUCILAIR medium was added to the cells, which were then incubated for 20 min (34 °C, 5% CO_2_, and 100% relative humidity). Apical washes were collected and stored at –80 °C until the quantification of genome copies or viral titrations. Non-coated samples (Non coated) and directly infected cultures (Control no-mask) were included as controls. Epithelial integrity during the experiment was monitored by measuring TEER. Non-treated, non-infected MUCILAIR cultures (Mock) and cultures treated with the detergent Triton X-100 served as additional negative and positive control for TEER measurement. The medium under MUCILAIR inserts wells was changed once 48 hpi with 500 µL fresh MUCILAIR culture medium.

### Saline protocol

Salt solutions at concentrations of 0.9%, 3.5%, and 35% were prepared in demineralized water. An A/H3N2 virus solution (5 µL) containing 10^6^ genome copies was diluted 1:1 with MUCILAIR culture medium (Control Medium) or with the various saline solutions and incubated at room temperature for 10 min. The control medium and saline solutions containing the virus were diluted 1:10 in MUCILAIR culture medium and 100 µL of the dilutions was inoculated apically onto MUCILAIR inserts (n = 3) for infection for 3 h. Viruses were collected and quantified at 3.5, 24, 72, and 144 h post infection. Epithelial integrity during the experiment was monitored by measuring TEER.

### Epithelial integrity assessment and viral quantification

MUCILAIR epithelium integrity was assessed at 24, 72, and 144 h post infection by measuring TEER^32,33^ (EVOMX volt-ohm-meter, World Precision Instruments, Stevenage, UK).

Viral replication was assessed at 3.5, 24, 72, and 144 h post infection by the quantification of genome copies using TAQMAN (ThermoFisher Scientific, Waltham, MA, USA) probe RT-qPCR. Both methods are described in the Supplementary Information.

### Cell culture-based viral titrations (TCID_50_ assay)

We used MDCK-SIAT1 cells for viral titration (Merck). This cell line was established from the stable transfection of MDCK cells with cDNA encoding human α-2,6-sialyltransferase (SIAT1) to over-express 6-linked sialic acids^45^. The cells were cultured in growth medium consisting of Dulbecco’s modified Eagle’s medium with GlutaMAX (31,966,021; Life Technologies, ThermoFisher Scientific) supplemented with 10% (v/v) fetal calf serum (2-01F16-I; BioConcept Ltd, Allschwil/Basel, Switzerland), 1% non-essential amino acids (10,938,025; Life Technologies, ThermoFisher Scientific), and 1% penicillin–streptomycin (15,140,122; Life Technologies, ThermoFisher Scientific). The serum-free infection medium was 1% penicillin–streptomycin in Dulbecco’s modified Eagle’s medium, and the assay medium was serum-free medium supplemented with 1 µg/mL trypsin (T1426; Merck). Cultures were maintained at 37 °C in a 5% CO_2_ atmosphere. Confluent monolayers were washed with phosphate-buffered saline, and serum-free medium was added. The cells were inoculated in quadruplicate with tenfold serial dilutions of a virus solution and were incubated at 37 °C for 1 h for infection. Then, the inoculum was aspirated, the assay medium was added, and the cells were incubated at 37 °C. Four days post infection, the presence of cytopathic effect, visible as the detachment of dead cells from the monolayer, was observed and quantified under a light microscope. Apical washes of A/H3N2-infected MUCILAIR nasal epithelia collected at 72 h post infection were used for virus titration and quantification of genome copies for the following test conditions: Spr S1, Spr S3, Spr S10, Dip Dil10 × , Control no mask, Non coated, and Tween Str3. Viral titers were determined by the endpoint method of Reed and Muench^46^ and expressed as log_10_ TCID_50_/mL.

### Statistical analysis

Genome copy numbers and viral titers are log-normal distributed^47–49^. A few scientifically sensible comparisons were planned in the study design: log_10_-based genome copy numbers and viral titers in the non-coated control were compared with salt- and Tween-coated treatments by two-sample Student’s *t*-test with Welch modification to the degrees of freedom (one-tailed, R *t*-test function)^50^. The null hypothesis was that the true means in non-coated samples and with salt- and Tween-coated treatments were equal, and the alternative hypothesis was that true means in samples with coated treatments were smaller than that in non-coated control. *P*-values < 0.05 were considered significant and led to the rejection of the null hypothesis.

## Supplementary Information

Supplementary Information.

## Data Availability

The datasets generated and analyzed in the present study are available from the corresponding author on reasonable request.
