# Supplementary material for: In vitro testing of salt coating of fabrics as a potential antiviral agent in reusable face masks
Source: Sci Rep. 2022 Oct 11;12:17041. doi: 10.1038/s41598-022-21442-7 (PMC9552714; doi:10.1038/s41598-022-21442-7)
Supplement: Supplementary file 1 — Supplementary Information. [file 41598_2022_21442_MOESM1_ESM.pdf]

# *In vitro* testing of salt coating of fabrics as a potential antiviral agent in reusable face masks

Sandra Schorderet Weber[1*], Xavier Bulliard[2*], Rosy Bonfante[3*], Yang Xiang[1], Silvia Biselli[2], Sandro Steiner[1], Samuel Constant[3], Raphael Pugin[2], Alexandra Laurent[1], Shoaib Majeed[1], Stefan Lebrun[1], Michele Palmieri[2], Andreas Hogg[4], Arkadiusz Kuczaj[1], Manuel C. Peitsch[1], Julia Hoeng[1], Adrian Stan[1]

[1]PMI R&D, Philip Morris Products S.A., Quai Jeanrenaud 5, CH-2000 Neuchâtel, Switzerland
[2]Centre Suisse d'Electronique et de Microtechnique SA (CSEM), Rue Jaquet-Droz 1, CH-2002 Neuchâtel, Switzerland
[3]Epithelix Sàrl, Chemin des Aulx 18, CH-1228 Plan-les-Ouates, Geneva, Switzerland
[4]Coat-X SA, Eplatures-Grise 17, CH-2300 La Chaux-de-Fonds, Switzerland

*These authors contributed equally.
Corresponding author: Sandra.SchorderetWeber@contracted.pmi.com

# Supplementary Information

## Materials and Methods

### Virus stocks

Viruses from the same stocks were used in experiments without an additional passage. Prior to infection, the apical side of MucilAir™ cultures was washed once for 10 min and inoculated with 100 µL of culture medium containing $10^5$ virus particles (virus concentration, $10^6$ genome

copies/mL) for 3 h at 34 °C in the presence of 5% $CO_2$ under 100% relative humidity. After the incubation, unbound viruses were washed away by three rapid washes with the culture medium. Residual viruses were collected by a 20-min apical wash and were quantified by quantitative reverse-transcription PCR (RT-qPCR; QuantiTect Probe RT-PCR; Qiagen, Hilden, Germany) to establish a baseline for viral replication at later time points. New virus particles were collected by 20-min apical washes at 24, 72, and 144 h post infection. Viruses produced over several days were pooled, quantified by RT-qPCR, aliquoted, and stored at –80 °C until use.

**Scanning electron microscopy**

Salt crystals on the fabrics were visualized by scanning electron microscopy (FEI Scios2; Thermo Fisher Scientific, Waltham, MA, USA) in low vacuum mode. The presence of salt impregnated in the fabric fibers was confirmed using energy-dispersive X-ray spectroscopy using an X-Max 50 $mm^2$ EDS Detector (Oxford Instruments, High Wycombe, UK) and the AZtec software.

**MucilAir epithelium quality check**

Airway epithelium is a layer of pseudostratified epithelium. When reconstituted on a semiporous membrane, epithelia have a homogenous and uniform appearance. As Transwell® membranes (Corning, Glendale, AZ, USA) are clear and transparent, morphological changes in the cells grown on them can easily be monitored. Each insert was inspected under a conventional inverted microscope (Leica DMIRE2; Leica Microsystems CMS GmbH, Mannheim, Germany) to ensure the quality of the epithelia. The homogeneity and uniformity of the pseudostratified airway epithelia were inspected. Ciliary movement was clearly visible in all selected inserts, and the presence of mucus was detected by the refractive aspect of the apical surface.

All inserts used in the experiments were washed apically with culture medium 3 d before the experiments to remove accumulated mucus and cell debris and thus minimize the risk of interference with the toxicity tests. The transepithelial electrical resistance (TEER) was also measured to verify that all selected inserts satisfied the internal quality control standards (TEER > 200 $\Omega.cm^2$).

To check the integrity of the epithelia throughout the study, three selected inserts (mock) were not infected but exposed to 100 μL of culture medium on the apical side for 3 h and handled in the same way as the virus-exposed inserts. To one insert, Triton X-100 (100 μL of a 10% v/v solution in 0.9% saline) was added as a control of maximum epithelial disruption.

**TEER measurement**

For TEER measurements, 200 μL of buffered saline solution was added to the apical compartment of MucilAir cultures and resistance was measured using an EVOMX volt-ohm-meter (World Precision Instruments, Stevenage, UK). Resistance values (Ω) were converted to TEER values ($\Omega{\cdot}cm^2$) using the following formula: TEER ($\Omega{\cdot}cm^2$) = (resistance value (Ω) − 100(Ω)) × 0.33 ($cm^2$), where 100 Ω is the resistance of the membrane and 0.33 $cm^2$ is the total surface of the epithelium.

**Real-time TaqMan® probe RT-qPCR**

From the 200-μL apical washes, 100 μL was used for viral RNA extraction using the Quick-DNA/RNA™ Viral kit (Zymo Research, Irvine, CA, USA) into a final elution volume of 50 μL. The viral RNA (in 5 μL of viral RNA extract) was quantified by RT-qPCR (QuantiTect Probe RT-PCR; Qiagen, Hilden, Germany) using TaqMan Master Mix, a pair of influenza A-specific primers (forward: 5' GAC CRA TCC TGT CAC CTC TGA C 3'; reverse: 5' AGG GCA TTY TGG ACA AAK CGT CTA 3'), and an influenza A probe (5' TGC AGT CCT CGC TCA CTG GGC ACG 3') with FAM–TAMRA reporter–quencher dyes. Four dilutions of a known concentration of A/H3N2 as well as of controls for RNA extraction and RT-qPCR were included, and the reactions were run on a Chromo4 PCR Detection System from Bio-Rad (http://eqdb.nrf.ac.za/equipment-make/bio-rad; Hercules, CA, USA). The limit of quantification of the method was established at 390 gc/mL. Ct values were converted into RNA copy numbers per reaction using the slope-intercept form, corrected with the dilution factor applied during viral RNA extraction, and presented as $\log_{10}$ genome copy numbers per milliliter in graphs.

# Results

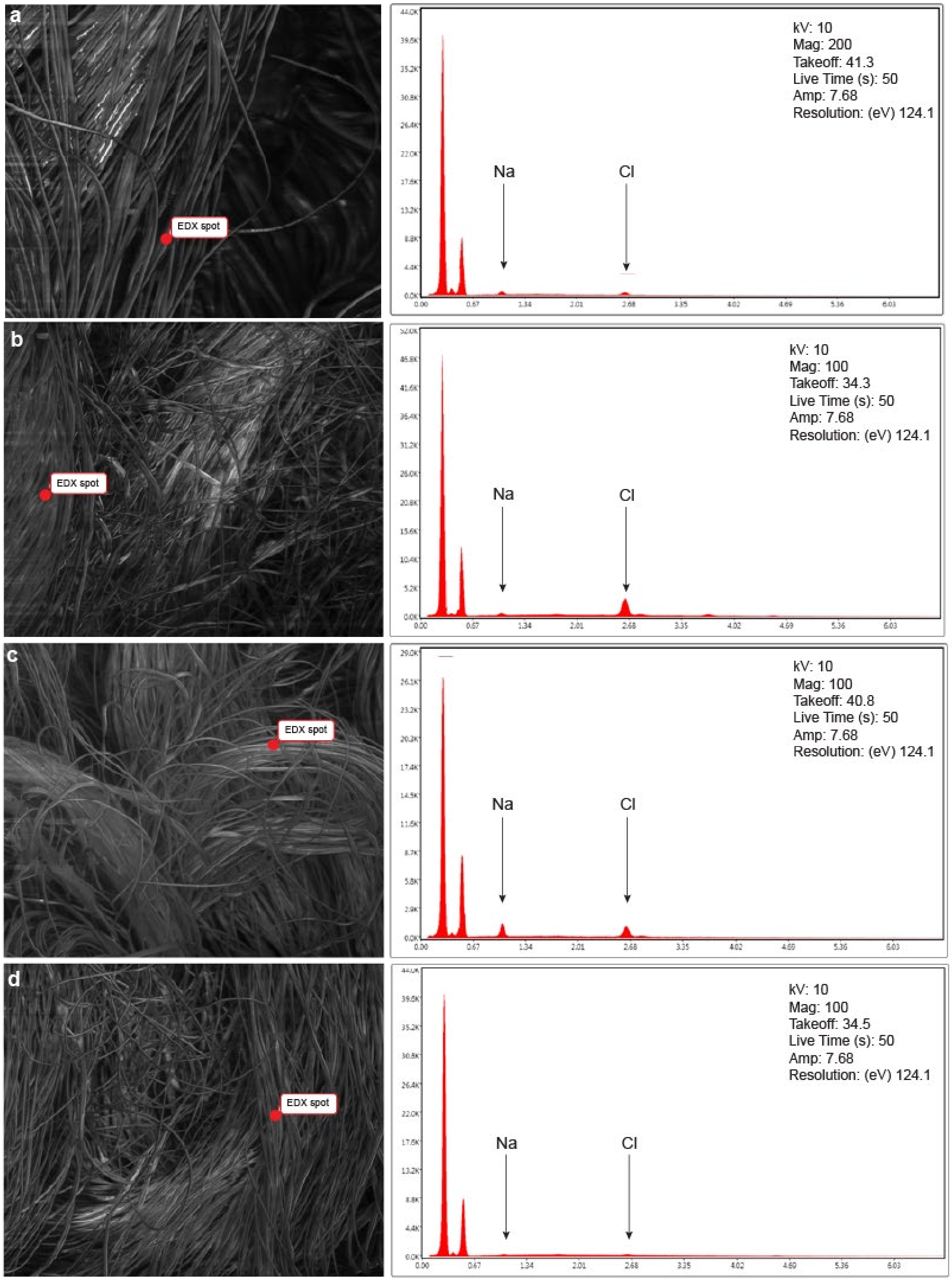
a
EDX spot
kV: 10
Mag: 200
Takeoff: 41.3
Live Time (s): 50
Amp: 7.68
Resolution: (eV) 124.1
Na
Cl
b
EDX spot
kV: 10
Mag: 100
Takeoff: 34.3
Live Time (s): 50
Amp: 7.68
Resolution: (eV) 124.1
Na
Cl
c
EDX spot
kV: 10
Mag: 100
Takeoff: 40.8
Live Time (s): 50
Amp: 7.68
Resolution: (eV) 124.1
Na
Cl
d
EDX spot
kV: 10
Mag: 100
Takeoff: 34.5
Live Time (s): 50
Amp: 7.68
Resolution: (eV) 124.1
Na
Cl
0.00 0.67 1.34 2.01 2.68 3.35 4.02 4.69 5.36 6.03

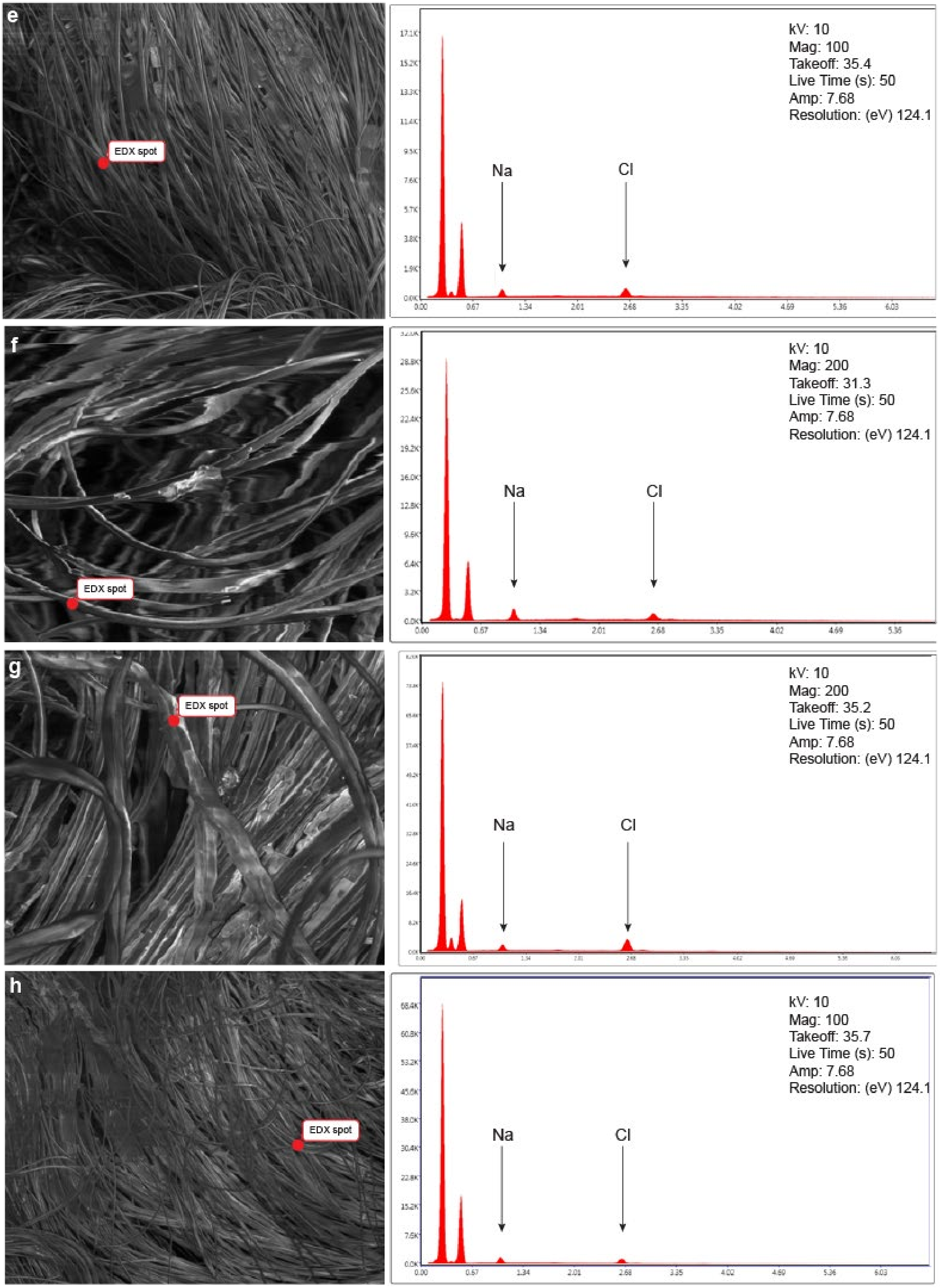

e
EDX spot
Na
Cl
kV: 10
Mag: 100
Takeoff: 35.4
Live Time (s): 50
Amp: 7.68
Resolution: (eV) 124.1
f
EDX spot
Na
Cl
kV: 10
Mag: 200
Takeoff: 31.3
Live Time (s): 50
Amp: 7.68
Resolution: (eV) 124.1
g
EDX spot
Na
Cl
kV: 10
Mag: 200
Takeoff: 35.2
Live Time (s): 50
Amp: 7.68
Resolution: (eV) 124.1
h
EDX spot
Na
Cl
kV: 10
Mag: 100
Takeoff: 35.7
Live Time (s): 50
Amp: 7.68
Resolution: (eV) 124.1

**Fig. S1.** Energy-dispersive X-ray spectroscopy of spray and dip-coated fabric fibers. Spray treatments allowed the deposition of increasing amounts of salt by varying the valve aperture of the spray device according to arbitrary stroke units. (**a**) Spray, undiluted salt formulation, stroke unit 1 (Spr S1), (**b**) spray, 5-fold diluted salt formulation, stroke unit 3 (Spr S3 Dil5×), (**c**) spray, undiluted salt formulation, stroke unit 3 (Spr S3), (**d**) spray, undiluted salt formulation, stroke unit 5 (Spr S5), (**e**) spray, undiluted salt formulation, stroke unit 10 (Spr S10). For dip treatments test materials were immersed into (**f**) undiluted (Dip No Dil), (**g**) 5-fold diluted (Dip Dil5×), and (**h**) 10-fold diluted (Dip Dil10×) salt formulations. ● area of the test sample analyzed by energy-dispersive X-ray spectroscopy.

Energy-dispersive X-ray spectroscopy of the spray-coated samples Spr S1, Spr S3 Dil 5×, Spr S3, Spr S5, and Spr S10 (Fig. S3a–e, respectively), and dip-coated samples Dip No Dil, Dip Dil5×, and Dip Dil10× (Fig. S3f-h, respectively), confirmed that salt was present in areas of the test samples where crystals were not observed by scanning electronic microscopy.

**Table S1.** Salt concentrations in A/H3N2 incubation medium (saline test) or in A/H3N2 virus collection medium (salt coating test). For dip-coated samples, mean weight ($mg/cm^2$) ± standard deviation is presented, $n = 3$.

| | Test group | Initial salt concentration | Final salt concentration in virus-containing medium |
|---|---|---|---|
| | | mg/mL | mg/mL |
| Saline concentrations | 0.90% | 9.00 | 0.90 |
| | 3.50% | 35.00 | 2.20 |
| | 35% | 350.00 | 17.9 |
| | | $mg/cm^2$ | mg/mL |
| Salt concentration in the coating on the fabric | Stroke 1 | 0.58 | 0.96 |
| | Stroke 3 | 4.73 | 1.37 |
| | Stroke 3 Dil5× | 0.41 | 0.94 |
| | Stroke 5 | 6.49 | 1.55 |
| | Stroke 10 | 19.42 | 2.84 |
| | Dip No Dil | 45.54 ± 1.70 | 5.45 |
| | Dip Dil5× | 10.00 ± 0.98 | 1.90 |
| | Dip Dil10× | 4.34 ± 0.089 | 1.33 |
